# Evidence of metasomatism in the interior of Vesta

**DOI:** 10.1038/s41467-020-15049-7

**Published:** 2020-03-10

**Authors:** Ai-Cheng Zhang, Noriyuki Kawasaki, Huiming Bao, Jia Liu, Liping Qin, Minami Kuroda, Jian-Feng Gao, Li-Hui Chen, Ye He, Naoya Sakamoto, Hisayoshi Yurimoto

**Affiliations:** 1 State Key Laboratory for Mineral Deposits Research and School of Earth Sciences and Engineering, Nanjing University, Nanjing, 210023 China; 2 CAS Center for Excellence in Comparative Planetology, Hefei, China; 3 Department of Natural History Sciences, Hokkaido University, Sapporo, 060-0810 Japan; 4 Department of Geology & Geophysics, Louisiana State University, Baton Rouge, LA 70803 USA; 5 International Center for Isotope Effects Research, Nanjing University, Nanjing, 210023 China; 6 School of Earth Sciences and Engineering, Nanjing University, Nanjing, 210023 China; 7 CAS Key Laboratory of Crust-Mantle Materials and Environment, University of Science and Technology of China, Hefei, 230026 China; 8 State Key Laboratory of Ore Deposit Geochemistry, Institute of Geochemistry, Chinese Academy of Sciences, Guiyang, 550081 China; 9 Isotope Imaging Laboratory, Creative Research Institution, Hokkaido University, Sapporo, 001-0021 Japan; 10 Institute of Space and Astronautical Science, Japan Aerospace Exploration Agency, Kanagawa, 252-5210 Japan

**Keywords:** Asteroids, comets and Kuiper belt, Meteoritics, Petrology

## Abstract

Diogenites are a group of meteorites that are derived from the interior of the largest protoplanet Vesta. They provide a unique opportunity to understanding together the internal structure and dynamic evolution of this protoplanet. Northwest Africa (NWA) 8321 was suggested to be an unbrecciated noritic diogenite meteorite, which is confirmed by our oxygen and chromium isotopic data. Here, we find that olivine in this sample has been partly replaced by orthopyroxene, troilite, and minor metal. The replacement texture of olivine is unambiguous evidence of sulfur-involved metasomatism in the interior of Vesta. The presence of such replacement texture suggests that in NWA 8321, the olivine should be of xenolith origin while the noritic diogenite was derived from partial melting of pre-existing rocks and had crystallized in the interior of Vesta. The post-Rheasilvia craters in the north-polar region on Vesta could be the potential source for NWA 8321.

## Abstract (web summary)

The authors here analyse the petrology of the meteorite NWA 8321 (parent body Vesta). They find sulfidation processes of olivine suggesting metasomatism in the Vestan interior and a partial melting origin for the host noritic diogenite.

## Introduction

Asteroid 4-Vesta is one of the largest celestial bodies in the main asteroid belt between Mars and Jupiter. Recent observations based on the Dawn mission revealed that Vesta is the largest protoplanet with a core-mantle-crust structure^1^. Its internal structure and evolutionary history has significant implications for understanding the formation and early evolution of differentiated celestial bodies in the solar system, including the Moon and terrestrial planets. Mineralogical analysis based on reflectance spectra confirmed that asteroid 4-Vesta could be the parent body of the howardite-eucrite-diogenite (HED) meteorites^1–3^. Thus, HED meteorites can potentially provide detailed information about the internal structure and evolution of this largest differentiated protoplanet.

The HED meteorites have various mineral assemblages, probably representing materials from different depths of Vesta^3,4^. Eucrites that consist mainly of pyroxene and plagioclase are thought being derived from the upper crust of Vesta. Diogenites consist mainly of orthopyroxene with or without olivine. They are usually coarse-grained and have Mg/(Mg + Fe) values higher than eucrites, suggesting that they were derived from the lower crust or the mantle of Vesta, based on magma ocean models^3–8^. Howardites are mechanical mixtures of eucrite and diogenite components, representing materials from the surface of Vesta. As materials from the Vestan interior, diogenites are critical to constraining the internal structure and dynamic evolution of the interior of Vesta and to testing the magma ocean models for Vesta. However, origins of diogenites are still an issue of controversy^9,10^. The recent magma ocean model^7^ claimed that equilibrium crystallization followed by fractional crystallization can produce all of the igneous HED lithologies. However, a few investigations suggested that the large variations in incompatible trace element concentrations among diogenites are difficult to be explained by the magma ocean models^9–13^. Some diogenites contain glassy materials and chemically zoned orthopyroxene, also indicating that the origins of diogenites could be very complex^11,14^. Based on the large variations in incompatible trace element concentrations and the presence of chemically zoned orthopyroxene, a number of investigations^10–14^ suggested that many diogenites were formed through partial melting of the magma ocean cumulates, probably with contamination of melts derived from anataxis of eucritic crust. However, considering the potential effects from post-magmatic metamorphism on the eucrite parent body, terrestrial weathering, and analytical issues, whether partial melting had taken place in Vesta remains an open question^9^.

Northwest Africa (NWA) 8321 is an unbrecciated noritic diogenite meteorite^15^. In this noritic diogenite, we find a unique replacement texture of olivine by troilite, orthopyroxene, and minor metal. The presence of replacement texture not only indicates metasomatism in the interior of Vesta but also suggests that noritic diogenite should be derived from partial melting of pre-existing rocks.

## Results

### Oxygen and chromium isotope compositions

The NWA 8321 meteorite was classified as a diogenite originally based only on its petrography and mineral compositions^15^. In the current study, our bulk oxygen and chromium isotope compositions (Δ^17^O_SMOW_ = –0.238 ± 0.017‰; ε^54^Cr = –0.58 ± 0.11; Supplementary Fig. 1) confirm that NWA 8321 shares a common parent body with typical HED meteorites.

### Petrography and mineralogy

**Fig. 1:**
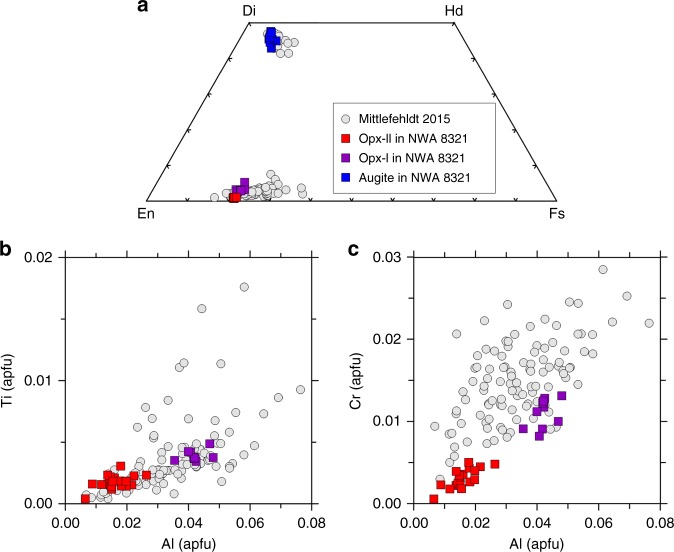
Chemical compositions of orthopyroxene and augite in NWA 8321. **a** quadrilateral diagram showing the chemical compositions of major elements in pyroxene. Ti vs. Al (**b**) and Cr vs. Al (**c**) in orthopyroxene. Literature data from ref. ^4^ are also plot for comparison.

**Fig. 2:**
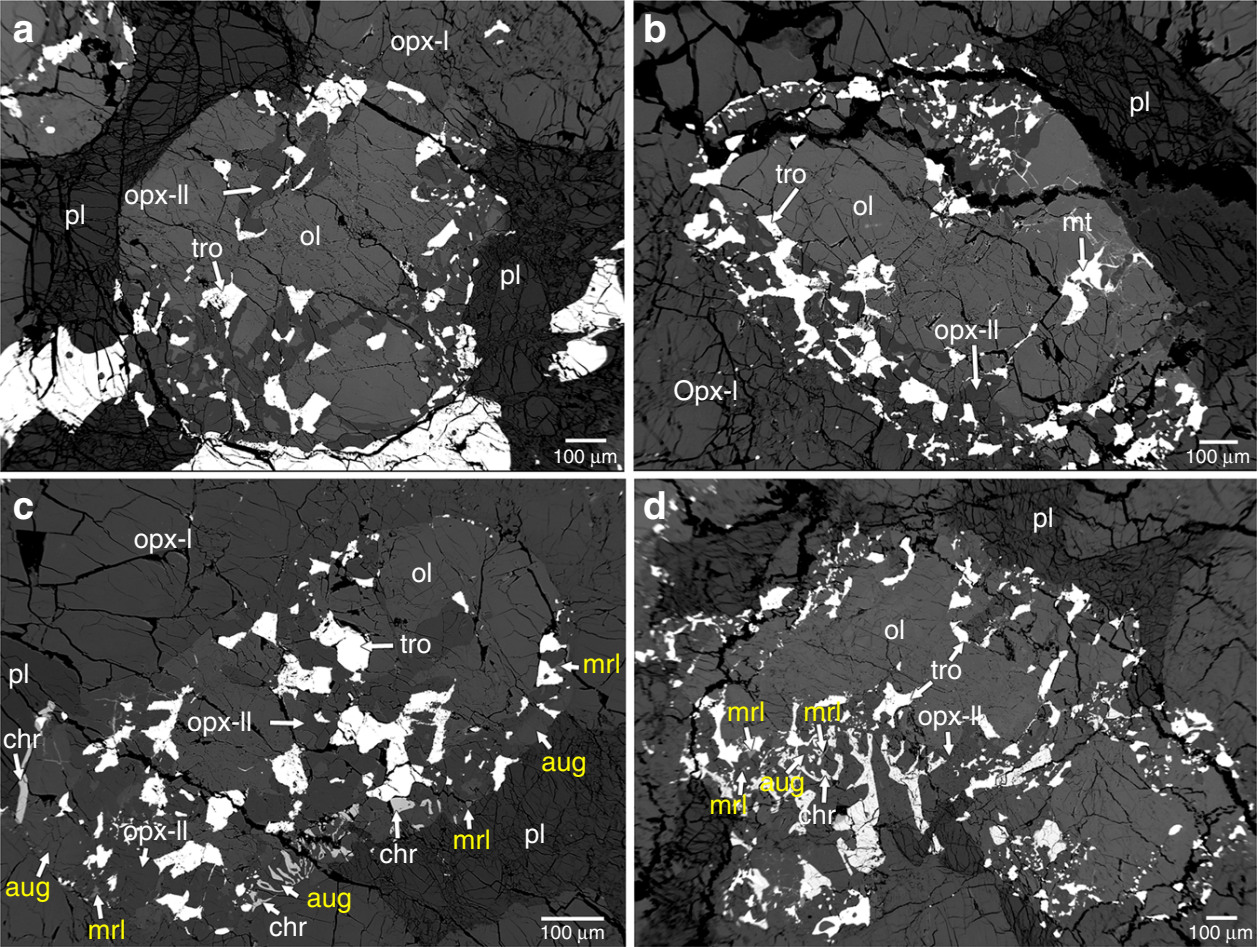
Backscattered electron images showing the replacement texture of olivine in NWA 8321. **a**, **b** Troilite and orthopyroxene form at the margin and in interior of an euhedral olivine grain. Note FeNi metal is also closely associated with troilite and orthopyroxene in (**b**). **c**, **d** Fine-grained augite, chromite, and merrillite are present, surrounding the olivine grains. ol: olivine; pl: plagioclase; aug: augite; mrl: merrillite; chr: chromite; opx: orthopyroxene; tro: troilite; mt: metal.

The NWA 8321 meteorite consists mainly of orthopyroxene (~84 vol%) and plagioclase (~10–12 vol%) with minor olivine, chromite, troilite, augite, merrillite, and ilmenite (Supplementary Fig. 2). The orthopyroxene grains are subhedral to euhedral in shape and coarse-grained (1–4 mm in size; labeled as Opx-I hereafter). Their Mg# values [Mg/(Mg+Fe) in mole] are 0.77–0.80 and the Fe/Mn values are 25–27. The concentrations of Ca, Al, Cr, and Ti in Opx-I are similar to those of typical diogenitic orthopyroxene (Fig. 1; ref. ^4^). Plagioclase in NWA 8321 occurs as an interstitial phase among orthopyroxene grains and is anorthitic (An_88–92_). Olivine (Mg# = 0.76–0.77) is commonly observed in NWA 8321 but has a low and variable modal abundance (<3 vol%) in different polished sections. Distinct from olivine in the most diogenites reported in the literature^12,16–21^, most of the olivine grains in NWA 8321 are closely associated with orthopyroxene (labeled as Opx-II hereafter), troilite, and minor FeNi metal (<2 wt% Ni). The Opx-II and troilite occur either at the margin of olivine grains or penetrate into the interior of olivine grains (Fig. 2a, b). Opx-II and troilite have an area ratio of ~2–3. The olivine + Opx-II + troilite assemblage usually has a rounded to smoothed outline. Many of the olivine + Opx-II + troilite mineral assemblages are partly or entirely included in Opx-I (Supplementary Fig. 2). Opx-II has Mg# compositions (0.78–0.79) and Fe/Mn values (25–29) similar to those of Opx-I. However, its concentrations of Ca, Al, Cr, and Ti are distinctly lower than those in Opx-I (Fig. 1).

A few coarse chromite grains (Chr_63.0–65.0_Spl_32.3–34.6_Usp_2.4–2.7_) are observed with a grain size of >1 mm. Some of them have a subhedral to anhedral shape which is confined to the shapes of olivine or orthopyroxene grains (Fig. 2a). Scanning electron microscopic observations also show that relatively small (tens of micrometers in size) and irregular grains of augite (En_45.9–48.1_Fs_6.5–9.1_Wo_42.9–47.6_; 0.40–1.36 wt% Al_2_O_3_ and 0.13–0.92 wt% Cr_2_O_3_), merrillite, and chromite (Chr_64.3–72.6_Spl_26.0–33.5_Usp_1.5–2.5_) are present at the margins of or around some of the olivine + Opx-II + troilite assemblages (Fig. 2c, d). In some regions, fine-grained chromite and augite form a symplectite texture (Fig. 2c, d). One subhedral ilmenite grain was observed in this study, being closely associated with relatively fine-grained chromite and troilite. All of the chromite grains in NWA 8321 contain Ti concentrations much lower than the chromite and ulvöspinel from eucrites and comparable to the chromite from typical diogenites (Supplementary Fig. 3; ref. ^4^). Augite and spatially associated Opx-I indicate a two-pyroxene equilibrium temperature at 909 ± 30 ^o^C (ref. ^22^).

### Rare-earth element (REE) compositions of minerals

REE concentrations in silicate and phosphate minerals were determined using secondary ion mass spectrometry. Multiple measurements on Opx-I show consistent heavy-REE concentrations (Lu 2–3 × CI) but variable light-REE concentrations and negative Eu anomalies (Eu/Eu* = 0.04–0.38; Eu* ≡ (Sm + Gd)/2; Supplementary Fig. 4). The elevated concentrations of LREE (especially La, Ce, and Pr) and Eu in some of the Opx-I grains could be due to terrestrial alteration^23–25^. Differing from Opx-I, the REE concentrations in Opx-II are below detection limit (Lu <0.5 × CI). The REE concentrations in olivine are below detection limit as well. Merrillite and discrete augite grains have different REE concentrations (Lu 1800 × CI for merrillite and 7–11 × CI for augite) but similar REE patterns, which are middle-REE-rich and contain a deeply negative Eu anomaly (Eu/Eu* = 0.07 for merrillite; Eu/Eu* = 0.06–0.10 for augite). Plagioclase shows a light-REE-enriched pattern (La 2.2–6.3 × CI with heavy-REE below detection limit) with a strongly positive Eu anomaly (Eu/Eu* = 120–360).

### Whole-rock composition and MELTS calculations

The whole-rock composition of major elements in NWA 8321 is given in Supplementary Table 7. Using this whole-rock composition, potential crystallization sequences are calculated at various crystallization pressures from 0.4 to 0.01 GPa and an oxygen fugacity of FQM-5 using the Rhyolite-MELTS software^26^. The Rhyolite-MELTS calculations at various pressures (Supplementary Fig. 5) give generally similar crystallization phases and sequences, except for olivine and ilmenite. In general, spinel, orthopyroxene, and metal are the early phases crystallized from the melt and have a large temperature interval of crystallization; while clinopyroxene, feldspar, and whitlockite are late-stage crystallization phases. At a crystallization pressure >0.1 GPa, olivine crystallizes relatively late, at a temperature slightly above 1200 ^o^C. However, at a pressure ≤ 0.1 GPa, olivine crystallizes at two different temperature intervals (Supplementary Figs. 5b–d). Ilmenite is absent at a pressure >0.06 GPa but becomes a late-stage phase from the melt at pressure ≤ 0.06 GPa (Supplementary Fig. 5).

## Discussion

One of the most striking petrographic features in NWA 8321 is that most of the olivine grains are closely associated with troilite, Opx-II, and minor metal. The troilite, Opx-II, and metal assemblage either occurs at the margin of olivine grains or penetrates into the interior of olivine grains. Similar textures have been reported not only in a few brecciated HED meteorites^25,27,28^, but also in Apollo samples^29^ and an olivine-rich primitive achondrite Divnoe^30^. Two hypotheses have been proposed to interpret this texture. One hypothesis suggested that the intergrowing orthopyroxene and troilite were formed by shock-induced melting and recrystallization of pre-existing orthopyroxene and troilite^28^. In this hypothesis, the formation of the intergrowing orthopyroxene and troilite was not related to olivine. The other hypothesis suggested that the intergrowing orthopyroxene and troilite are products of the replacement reaction (sulfidation) between olivine and S-rich medium in an open system^25,27,29,30^. In the current study, Opx-II and troilite are closely associated with irregular olivine, implying that olivine was replaced by Opx-II and troilite. Meanwhile, Opx-II in NWA 8321 contains lower concentrations of Ca, Al, Cr, Ti, and REE than Opx-I, which can be best interpreted by a reaction between olivine and S-rich medium: Ca, Al, Cr, Ti, and REE are usually more incompatible in olivine than in orthopyroxene, orthopyroxene grains that formed via replacing olivine are expected to have retained this depletion feature^25,27,30^. Therefore, both textural and chemical features indicate that the olivine grains in NWA 8321 were partly replaced by troilite, orthopyroxene, and minor metal. The first hypothesis involving shock-induced melting and recrystallization of pre-existing orthopyroxene and troilite is difficult to explain the relative depletion of Ca, Al, Cr, Ti, and REE in Opx-II and can therefore be excluded. Given the presence of metal and troilite rather than pyrite, both the oxygen fugacity and the sulfur fugacity should be relatively low^25,31^. Combining the apparent compositions of relict olivine and Opx-II, the sulfidation of olivine could be Mg_1.52_Fe_0.48_SiO_4_ + 0.135S_2_ = Mg_0.79_Fe_0.21_SiO_3_ + 0.27FeS + 0.135O_2_ + 0.73MgO (reaction 1) or 79Mg_1.52_Fe_0.48_SiO_4_ + 3S_2_ + 73SiO_2_ = 152Mg_0.79_Fe_0.21_SiO_3_ + 6FeS + 3O_2_ (reaction 2). Reaction 1 implies that some magnesium would be lost during the sulfidation whereas reaction 2 requires the addition of SiO_2_ besides of S_2_ (refs. ^25,29^). In reaction 2, the reaction products orthopyroxene and troilite have a ratio (~25), which is much larger (by one order of magnitude) than the ratio (2–3) of Opx-II and troilite in our observation. Instead, reaction 1 has a ratio of orthopyroxene and troilite comparable to our observation. Meanwhile, considering the difficulty that reaction 2 requires the existence of almost pure SiO_2_ melt/vapor (not involving Ca, Al, Cr, and Ti) in the interior of Vesta, reaction 1 which only requires mobility of S_2_ and MgO seems more likely to interpret the texture and compositions for the coexisting olivine, Opx-II, troilite, and metal^25,29^. Lost of some elements from the system during sulfidation of silicate minerals has been observed in the literature^29,32,33^.

A few types of metasomatism on Vesta have been reported based on investigations on HED meteorites^25,27,28,32–38^. However, most of the metasomatic textures were correlated to surface or subsurface processes on Vesta^32–38^. Zhang et al.^25^ have observed a similar replacement texture in a diogenite meteorite NWA 7183 and proposed that the sulfidation of olivine might have taken place in the interior of Vesta. However, the sample they studied is a brecciated diogenite and has experienced post-magmatic Fe-enrichment^25^. Both of the two facts increased the uncertainty of the interpretation about the location of sulfidation. Differing from these brecciated HED meteorites with metasomatic textures of olivine in the previous investigations^25,27^, the NWA 8321 meteorite in the current study is an unbrecciated diogenite. Meanwhile, the high Mg# values of mafic silicate minerals indicate that NWA 8321 has not experienced Mg-Fe interdiffusion on the Vestan surface where Fe-rich eucrites are usually dominant^25^. Therefore, the possibility that the sulfidation of olivine in NWA 8321 had taken place at or near the surface of Vesta can be eliminated. Instead, the sulfidation of olivine in NWA 8321 must have taken place in the interior of Vesta. The depth of the sulfidation is difficult to be constrained directly. However, the crystallization depth of NWA 8321 might provide a lower limit for the depth of the sulfidation of olivine in the interior of Vesta. Therefore, we estimate the crystallization depth of NWA 8321 from two aspects. First, the two-pyroxene equilibrium temperature is ~900 ^o^C, implying a depth of tens of kilometers^39^, although the exact temperature gradient structure for Vesta is unknown. Second, we estimate the crystallization depth of NWA 8321 based on the crystallization pressure. In this study, ilmenite is a late-stage crystallization phase at relatively low crystallization pressures (≤0.06 GPa) according to the Rhyolite-MELTS calculations. It would be absent if the crystallization of NWA 8321 had taken place at higher pressures. Therefore, we consider the crystallization pressure of NWA 8321 should be <0.06 GPa. A static pressure of 0.06 GPa corresponds to a depth of ~10 km in Vesta, assuming the pressure and depth having a linear correlation and given a gravity constant of 0.22 m/s^2^ and the density of HED meteorites of approximately 2800–3200 kg/m^3^ (ref. ^1^). A depth on a scale of 10 km indicates that the parent magma of NWA 8321 might have crystallized in the crust of Vesta. The location of the sulfidation might be deeper than this depth, either in the crust or in the upper mantle of Vesta. In summary, the replacement of olivine by orthopyroxene, troilite and metal in NWA 8321 represents an unambiguous petrologic record of metasomatism in the Vesta interior, which has not been established in the previous investigations on HED meteorites^4,25^. The existence of metasomatism in the Vestan interior implies that the internal evolution of Vesta is much more complex than the magma ocean models^4–7^.

If that the sulfidation of olivine had taken place in the interior of Vesta as discussed above, an external source for the sulfur-rich medium is unlikely^32,33^. Instead, the sulfur-rich medium should be derived from the Vestan interior, although its exact nature and source cannot be constrained based on the current observations. One possibility is that the sulfur-rich medium might have formed due to degassing of a sulfur-bearing magma during its intrusion^25,29^. Degassing of sulfur has been suggested based on the presence of ^34^S enrichment in some eucrite meteorites^40^ and consideration on the depletion of volatile siderophile elements in eucrites^41^.

The petrographic texture that some of the mineral assemblage olivine + Opx-II + troilite ± metal is included entirely within Opx-I indicates that the sulfidation of olivine should predate the crystallization of Opx-I. This implies that the olivine in NWA 8321 should be of exotic (xenolith) origin, probably from pre-existing olivine-rich rocks (dunite or harzburgite). One may argue that some of the olivine grains in NWA 8321 could be a liquidus phase as the Rhyolite-MELTS calculations demonstrate and then a S-rich medium reacted with the olivine grains during or postdating the crystallization of Opx-I. If the sulfidation of olivine had taken place during the crystallization of Opx-I from melt, Opx-II and Opx-I would form simultaneously and no textural and chemical differences (Ca, Cr, Al, and Ti) between them would be observed. This is in conflict with the observations in this study. For a potential sulfidation event (by reaction 1) after the crystallization of Opx-I, the volumes of solid reaction products would be approximately 1.3 times the volume of olivine that has been reacted^25,29^. The volume increase due to the sulfidation would cause the presence of radiating fractures in Opx-I from the boundary with the original olivine grains. However, this was not observed in our sample. Therefore, the possibility that the sulfidation had taken place after the crystallization of Opx-I can be excluded. In addition, if the olivine in NWA 8321 is a liquidus phase, no spatial correlation between olivine and relatively fine-grained chromite, augite, and phosphate shown in Fig. 2c, d would be expected, although the Rhyolite-MELTS calculations indicate that all of them could be late-stage phases. Combining the above considerations together, we suggest that most, if not all, of the olivine grains in NWA 8321 should be of xenolith origin. The presence of olivine xenoliths in noritic diogenite NWA 8321 suggests that it is neither a cumulate that directly crystallized from the Vestan magma ocean nor a residue of partial melting of magma ocean cumulate. Instead, it should be derived from partial melting of pre-existing ultramafic to mafic rocks. The high abundance of plagioclase in NWA 8321 compared to typical diogenites is generally consistent with the partial melting origin of pre-existing ultramafic to mafic rocks. Olivine xenoliths might also have been consumed by the reaction with the noritic melt to form orthopyroxene. However, the proportion of olivine xenoliths that have reacted with the noritic melt is unknown.

Partial melting is an important hypothesis that has been proposed to interpret the chemical features of many diogenite meteorites^9–14^. However, it is still an issue of controversy, given the uncertainty due to terrestrial weathering, analytical difficulty, parent body thermal metamorphism, appropriate partitioning coefficients of rare earth elements between orthopyroxene and the melts it crystallized from, and presence of potential anomalous diogenites^9^. The current observations based on the NWA 8321 noritic diogenite cannot provide evidence arguing for or against the partial melting hypothesis for typical plagioclase-poor diogenites. However, it demonstrates an independent record that partial melting might have taken place in the interior of Vesta, although the driving force of the partial melting remains unclear.

Our observations indicate that both the sulfidation of olivine and possible reaction between xenolith olivine and noritic melt consume olivine. This provides a potential opportunity to explain the non-detection of olivine-rich lithology at the south-pole impact basins of Vesta^25^. We performed a modeling with varying modal abundances of olivine and various proportions of sulfurized olivine (Supplementary Fig. 6). The result demonstrates that for olivine-rich lithology (dunite and harzburgite with >50% olivine) a minimum of approximately 50% olivine should have been sulfurized to make relict olivine undetectable (<25%) by the Dawn’s Visible and Infrared Spectrometer^42,43^. However, sulfidation of olivine would lead to high bulk Fe concentrations (>14.7 wt%; Supplementary Fig. 6). If the sulfidation of olivine were the major process that had reduced the modal abundance of olivine in the Vestan interior at the south-pole impact basins, anomalous high Fe concentrations would be expected and can be detected by the Dawn’s Gamma Ray and Neutron Detector (GRaND) instrument. However, no regions with anomalously large-scale high Fe concentrations were observed at the south-pole impact basins by the GRaND instrument^44,45^. Meanwhile, no plagioclase-rich diogenites were observed there^45,46^. Therefore, the sulfidation of olivine and the potential reaction between olivine and noritic diogenite is at least not the main cause of the non-detection of olivine-rich lithology at the south-pole impact basins on Vesta.

Recently, detailed comparisons between the bulk compositions of HED meteorites and Dawn GRaND data revealed that a group of Yamato diogenites (called type B diogenites) that are enriched in Fe and plagioclase were probably derived from the post-Rheasilvia craters in the north-polar region on Vesta^45,46^. The NWA 8321 meteorite in the current study is also a plagioclase-rich diogenite. However, the apparent bulk Fe concentration of NWA 8321 is lower than those of the type B diogenites. As demonstrated above, sulfidation of olivine can result in a large increase of Fe. If noritic diogenites contain more olivine grains and the olivine grains were largely sulfurized, they would have bulk chemistry comparable to Yamato type B diogenites. Considering that olivine distributes heterogeneously in NWA 8321, it is likely that NWA 8321 might have been derived from a region where olivine-rich noritic diogenite is common. If this is the case, the post-Rheasilvia craters in the north-polar region on Vesta might be the potential source for NWA 8321. Beck et al.^46^ suggested that the north-polar Type B plutonism may have been associated with the Rheasilvia impact event. If this is correct, the Rheasilvia impact event may have triggered the partial melting of pre-existing ultramafic to mafic rocks in the Vestan interior to produce noritic diogenites.

In summary, our observations reveal that olivine in the unbrecciated diogenite meteorite NWA 8321 has been partly replaced by orthopyroxene, troilite, and minor metal, indicating the presence of metasomatism (sulfidation) in the interior of Vesta. The petrographic observations further reveal that olivine in NWA 8321 should be of xenolith origin and probably partly consumed by a reaction with a noritic melt. The presence of the olivine xenoliths in NWA 8321 implies that the host noritic diogenite is neither a cumulate that directly crystallized from the Vestan magma ocean nor a residue of partial melting of magma ocean cumulate. Instead, the noritic diogenite should be a product of partial melting of pre-existing ultramafic to mafic rocks and crystallized in the crust of Vesta. The similarity in mineral assemblage between NWA 8321 and Type B diogenites in the literature and the effect of sulfidation of olivine on bulk Fe concentration suggests that the post-Rheasilvia craters in the north-polar region on Vesta might be the potential source for NWA 8321.

## Methods

NWA 8321 is an unbrecciated diogenite meteorite^15^. Its total weight is 317 g. In this study, four polished sections are used.

### Petrography and major element analysis

Petrographic observations and X-ray elemental mapping of NWA 8321 were performed using a Zeiss Supra 55 Field Emission scanning electron microscope (FE-SEM) at Nanjing University, Nanjing, China. Mineral compositions were measured using JEOL JXA 8100 electron probe micro-analyzer (EPMA) at Nanjing University. An accelerating voltage of 15 kV and a beam current of 20 nA were used for all minerals in this study. The measurement times for elemental peak and background are 20 and 10 s, respectively, for most elements, except for Na and K (10 and 5 s for peak and background measurements, respectively). Natural and synthetic standards were used for concentration calibration. All data were reduced with the ZAF (atomic number-absorption-fluorescence) procedure. Representative compositions are given in supplementary materials. Typical detection limit is <0.02 wt%. All the microprobe data are given in Supplementary Tables 1–5.

### REE analysis of minerals

The REE compositions of minerals were determined using Cameca IMS-6f instrument at Hokkaido University, Japan. The primary beam of O^─^ was accelerated with −13 keV and irradiated on the sample surface to a diameter of ~25 μm. The primary beam current is 6–10 nA. Kinetic energy filtering was used to reduce interferences from molecular ions by offsetting the sample acceleration voltage (–100 eV). The energy bandwidth was 20 eV. The exit slit was set to a mass resolving power of ~500. Relative sensitivity factors between secondary ion intensity and concentration for each REE (relative to Si for silicate or Ca for phosphate, respectively) were determined using the Takashima augite, for which REE contents have been well determined by instrumental neutron activation analysis^47^. A glass standard JB1-a was also measured before and after measurements on the angrite fragment to monitor the stability of measurements. The REE compositions are given in Supplementary Table 6. For measurements on olivine and some orthopyroxene (Opx-II) grains, the counts for most of the heavy REEs usually display a sawtooth-like variation (between 1.25E−1 and 1E−6) among different cycles. During data reduction, we used the average values of the 15 cycles to constrain the detection limit. For measurements on plagioclase, Tb and Lu always have very low counts (1E−6). Other heavy REEs have anomalous high counts. Since plagioclase is LREE-enriched, the interference from LREE on HREE could be still very large for plagioclase. Therefore, we did not report the HREE data for plagioclase in this study.

### Whole-rock chemical compositions

Whole-rock compositions of major elements in NWA 8321 were measured using a Thermo Scientific ARL 9900 X-ray fluorescence (XRF) spectrometry at Nanjing University. Two fragments with a total weight approximately 1.6 g were powdered in an agate mortar. One-gram powder was fused to make a glass plate in atmosphere with a Li_2_B_4_O_7_ flux with a sample/flux ratio of 1:11 in weight. According to the measured values of rock reference material (BHVO-2), the uncertainties (relative standard deviation) are about 1% for elements with concentrations >1 wt% and about 10% for elements with concentrations <1 wt%. The results are given in the Supplementary Table 7.

### Whole-rock oxygen isotope measurement

Whole-rock oxygen isotope composition of NWA 8321 was measured at Louisiana State University, using Thermo Scientific MAT253 isotope ratio mass spectrometer. The sample was silt to <2 mm sizes grains and treated with 3 M HCl for 24 h with periodical ultrasonicing before washing clean and dried. The triple oxygen isotope composition was measured on O_2_ generated by CO_2_-laser fluorination. The Δ^17^O value is calculated using δ^17^O-0.52δ^18^O, relate to Vienna Standard Mean Ocean Water.

### Whole-rock chromium isotope measurement

Whole-rock chromium isotope composition of NWA 8321 was measured at University of Science and Technology of China, Hefei, China, using a Triton Plus TIMS system (Thermo Fisher Scientific, Waltham, MA, USA). The sample processing and measurement procedure followed ref. ^48^.

## Supplementary information

Supplementary information

## Data Availability

The authors declare that the majority of the data supporting the findings of this study are available in the paper or supplementary materials. The unpublished data are available from the corresponding author upon request.

## References

[1] Russell CT (2012). Dawn at Vesta: testing the protoplanetary paradigm. Science.

[2] McCord TB, Adams JB, Johnson TV (1970). Asteroid Vesta: spectral reflectively and compositional implications. Science.

[3] McSween HY (2013). Dawn; the Vesta-HED connection; and the geologic context for eucrites, diogenites, and howardites. Meteorit. Planet. Sci..

[4] Mittlefehldt DW (2015). Asteroid (4) Vesta: I. The howardite-eucrite-diogenite (HED) clan of meteorites. Chem. der Erde.

[5] Righter K, Drake MJ (1997). A magma ocean on Vesta: core formation and petrogenesis of eucrites and diogenites. Meteorit. Planet. Sci..

[6] Ruzicka A, Snyder GA, Taylor LA (1997). Vesta as the howardite, eucrite and diogenite parent body: implications for the size of a core and for large-scale differentiation. Meteorit. Planet. Sci..

[7] Mandler BE, Elkins-Tanton LT (2013). The origin of eucrites, diogenites, and olivine diogenites: Magma ocean crystallization and shallow magma chamber processes on Vesta. Meteorit. Planet. Sci..

[8] Greenwood RC (2014). The oxygen isotope composition of diogenites: evidence for early global melting on a single, compositionally diverse, HED parent body. Earth Planet. Sci. Lett..

[9] Mittlefehldt DW, Beck AW, Lee CTA, McSween HY, Buchanan P (2012). Compositional constraints on the genesis of diogenites. Meteorit. Planet. Sci..

[10] Barrat JA, Yamaguchi A (2014). Comment on “The origin of eucrites, diogenites, and olivine diogenites: Magma ocean crystallization and shallow magma processes on Vesta” by B. E. Mandler and L. T. Elkins-Tanton. Meteorit. Planet. Sci..

[11] Mittlefehldt DW (1994). The genesis of diogenites and the HED parent body petrogenesis. Geochim. Cosmochim. Acta.

[12] Barrat JA, Yamaguchi A, Benoit M, Cotton J, Bohn M (2008). Geochemistry of diogenites: Still more diversity in their parental melts. Meteorit. Planet. Sci..

[13] Barrat JA, Yamaguchi A, Zanda B, Bollinger C, Bohn M (2010). Relative chronology of crust formation on asteroid 4-Vesta: Insights from the geochemistry of diogenites. Geochim. Cosmochim. Acta.

[14] Yamaguchi A, Barrat JA, Ito M, Bohn M (2011). Posteucritic magmatism on Vesta: Evidence from the petrology and thermal history of diogenites. J. Geophys. Res..

[15] Ruzicka, A., Grossman, J., Bouvier, A. & Agee, C. B. *The Meteoritical Bulletin, No. 103* (The Meteoritical Society, 2015).

[16] Beck AW, McSween HY (2010). Diogenites as polymict breccias composed of orthopyroxenite and harzburgite. Meteorit. Planet. Sci..

[17] Beck AW (2011). MIL 03443, a dunite from Asteroid 4 Vesta: evidence for its classification and cumulate origin. Meteorit. Planet. Sci..

[18] Yamaguchi A, Barrat JA, Shirai N, Ebihara M (2015). Petrology and geochemistry of Northwest Africa 5480 diogenite and evidence for a basin-forming event on Vesta. Meteorit. Planet. Sci..

[19] Lunning NG, McSween HY, Tenner TJ, Kita NT (2015). Olivine and pyroxene from the mantle of asteroid 4 Vesta. Earth Planet. Sci. Lett..

[20] Tkalcec BJ, Brenker FE (2015). Asteroidal processes recorded by polyphaser deformation in a harzburgitic diogenite NWA 5480. J. Struct. Geol..

[21] Hahn TM, Lunning NG, McSween HY, Bodnar RJ, Taylor LA (2018). Mg-rich harzburgites from Vesta: Mantle residua or cumulates from planetary differentiation?. Meteorit. Planet. Sci..

[22] Wells PRA (1977). Pyroxene thermometry in simple and complex systems. Contrib. Mineral. Petrol..

[23] Barrat JA, Gillet P, Lesourd M, Blichert-Toft J, Poupeau GR (1999). The Tahtahouine diogenite: mineralogical and chemical effects of sixty-three years of terrestrial residence. Meteorit. Planet. Sci..

[24] Crozaz G, Floss C, Wadhwa M (2003). Chemical alteration and REE mobilization in meteorites from hot and cold deserts. Geochim. Cosmochim. Acta.

[25] Zhang AC (2018). Origin and implications of troilite-orthopyroxene intergrowths in the brecciated diogenite Northwest Africa 7183. Geochim. Cosmochim. Acta.

[26] Gualda GAR, Ghiorso MS, Lemons RV, Carley TL (2012). Rhyolite-MELTS: A modified calibration of MELTS optimized for silica-rich, fluid-bearing magmatic systems. J. Petrol..

[27] Hewins, R. H. Fractionation and equilibration in diogenites. *Lunar Planetary Sci.* **12**, 445–447 (1981).

[28] Patzer A, McSween HY (2012). Ordinary (mesostasis) and not-so-ordinary (symplectites) late-stage assemblages in howardites. Meteorit. Planet. Sci..

[29] Shearer CK (2012). Origin of sulfide replacement textures in lunar breccias. Implications for vapor element transport in the lunar crust. Geochim. Cosmochim. Acta.

[30] Petaev MI (1994). The Divnoe meteorite: petrology, chemistry, oxygen isotopes and origin. Meteoritics.

[31] Toulmin P, Barton PB (1964). A thermodynamic study of pyrite and pyrrhotite. Geochim. Cosmochim. Acta.

[32] Zhang AC, Wang RC, Hsu WB, Bartoschewitz R (2013). Bartoschewitz, R. Record of S-rich vapors on asteroid 4 Vesta: sulfurization in the Northwest Africa 2339 eucrite. Geochim. Cosmochim. Acta.

[33] Wang SZ, Zhang AC, Pang RL, Li Y, Chen JN (2019). Possible records of space weathering on Vesta: case study in a brecciated eucrite Northwest Africa 1109. Meteorit. Planet. Sci..

[34] Mittlefehldt MW, Lindstrom MM (1997). Magnesian basalt clasts from the EET 92014 and Kapoeta howardites and a discussion of alleged primary magnesian HED basalts. Geochim. Cosmochim. Acta.

[35] Treiman AH, Lanzirotti A, Xirouchakis D (2004). Ancient water on asteroid 4 Vesta: evidence from a quartz veinlet in the Serra de Mage eucrite meteorite. Earth Planet. Sci. Lett..

[36] Barrat JA (2011). Possible fluid-rock interactions on differentiated asteroids recorded in eucritic meteorites. Geochim. Cosmochim. Acta.

[37] Mittlefehldt MW (2013). Composition and petrology of HED polymict breccias: the regolith of (4) Vesta. Meteorit. Planet. Sci..

[38] Warren PH (2014). Northwest Africa 5738: multistage fluid-driven secondary alteration in an extraordinary evolved eucrite. Geochim. Cosmochim. Acta.

[39] Yamaguchi A, Taylor GJ, Keil K (1997). Metamorphic history of the eucritic crust of 4 Vesta. J. Geophys. Res..

[40] Wu N, Farquhar J, Dottin III JW, Magalhães N (2018). Sulfur isotope signatures of eucrites and diogenites. Geochim. Cosmochim. Acta.

[41] Steenstra ES (2019). Significant depletion of volatile elements in the mantle of asteroid Vesta due to core formation. Icarus.

[42] Beck AW (2013). Challenges in detecting olivine on the surface of 4 Vesta. Meteorit. Planet. Sci..

[43] McSween HY (2013). Composition of the Rheasilvia basin, a window into Vesta’s interior. J. Geophys. Res..

[44] Yamashita N (2013). Distribution of iron on Vesta. Meteorit. Planet. Sci..

[45] Beck AW (2015). Using HED meteorites to interpret neutron and gamma-ray data from asteroid 4 Vesta. Meteorit. Planet. Sci..

[46] Beck AW (2017). Igneous lithologies on asteroid (4) Vesta mapped using gamma-ray and neutron data. Icarus.

[47] Onuma N, Higuchi H, Wakita H, Nagasawa H (1968). Trace element partition between 2 pyroxenes and host lava. Earth Planet. Sci. Lett..

[48] Qin L, Alexander CMOD, Carlson RW, Horan MF, Yokoyama T (2010). Contributors to chromium isotope variation of meteorites. Geochim. Cosmochim. Acta.

