# Supplementary material for: Evidence of metasomatism in the interior of Vesta
Source: Nat Commun. 2020 Mar 10;11:1289. doi: 10.1038/s41467-020-15049-7 (PMC7064581; doi:10.1038/s41467-020-15049-7)
Supplement: Supplementary file 1 — Supplementary information [file 41467_2020_15049_MOESM1_ESM.pdf]

Supplementary information for:

Evidence of metasomatism in the interior of Vesta

by Zhang et al.

Supplementary Figures

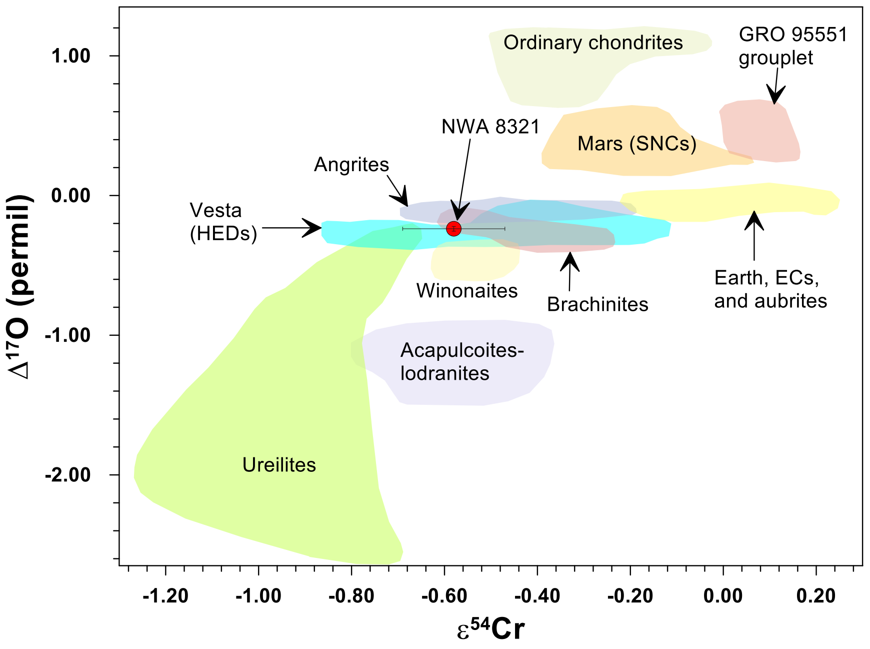

Supplementary Fig. 1 | Oxygen and chromium isotope compositions of NWA 8321 in comparison with other achondrite meteorites. The regions for different achondrite meteorites are adapted from Supplementary Ref. 1.

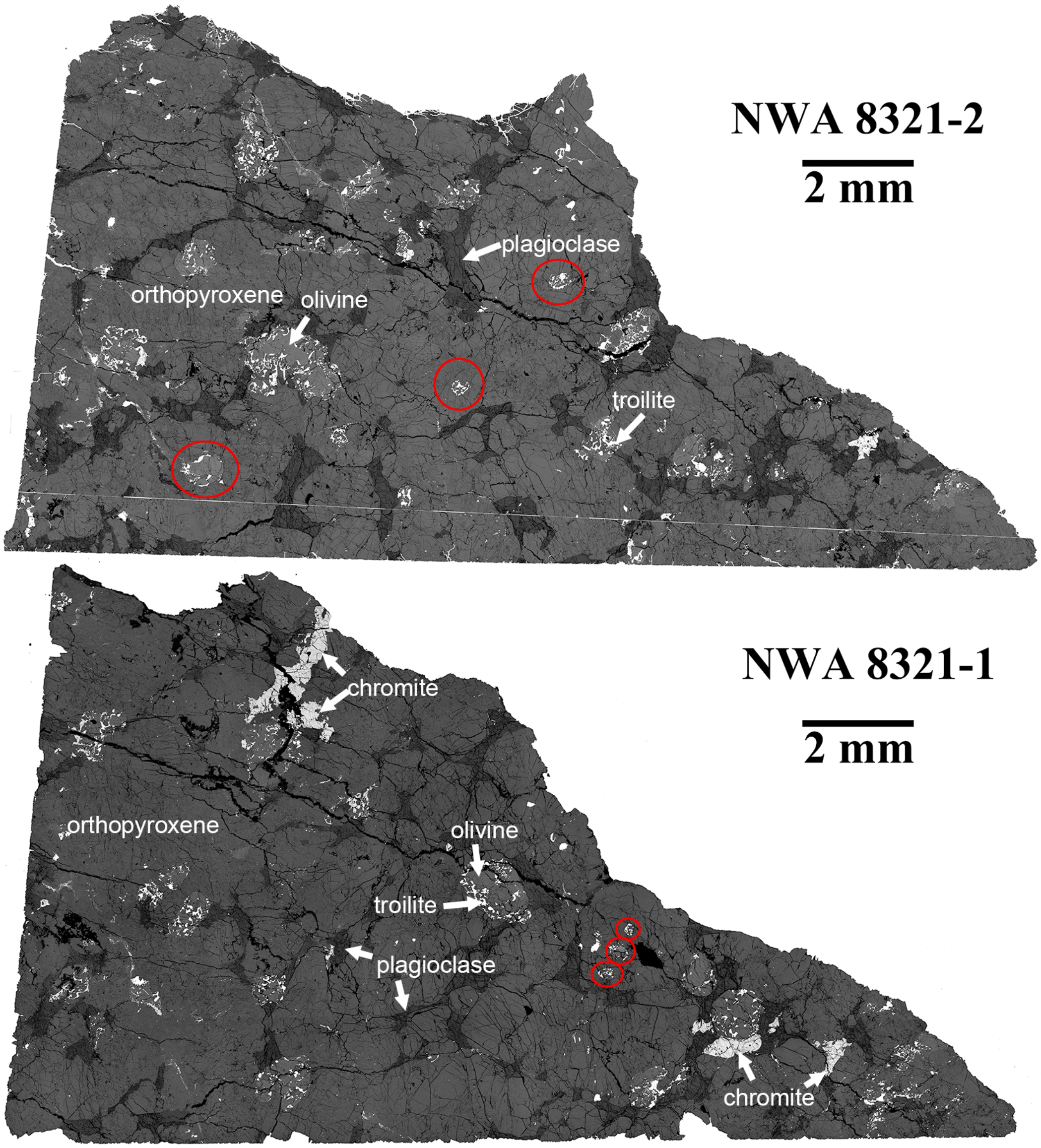

Supplementary Fig. 2 | Mosaic backscattered electron image of two sections of NWA 8321. Red circles indicate the olivine grains that are totally included in orthopyroxene (Opx-I).

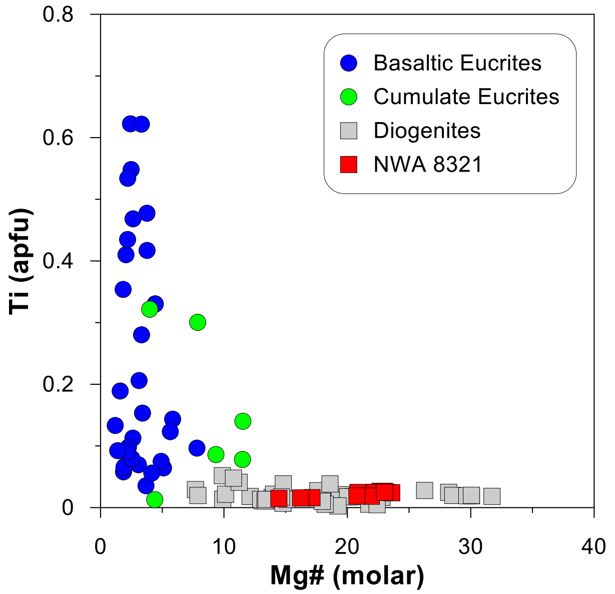

Supplementary Fig. 3 | Compositions of chromite in NWA 8321 in comparison with chromite and ulvöspinel in other HED meteorites. The red squares are chromite from NWA 8321. The grey squares, blue circles, and green circles are chromite and ulvöspinel in diogenite, basaltic eucrite, and cumulate eucrite from Ref. 4, respectively.

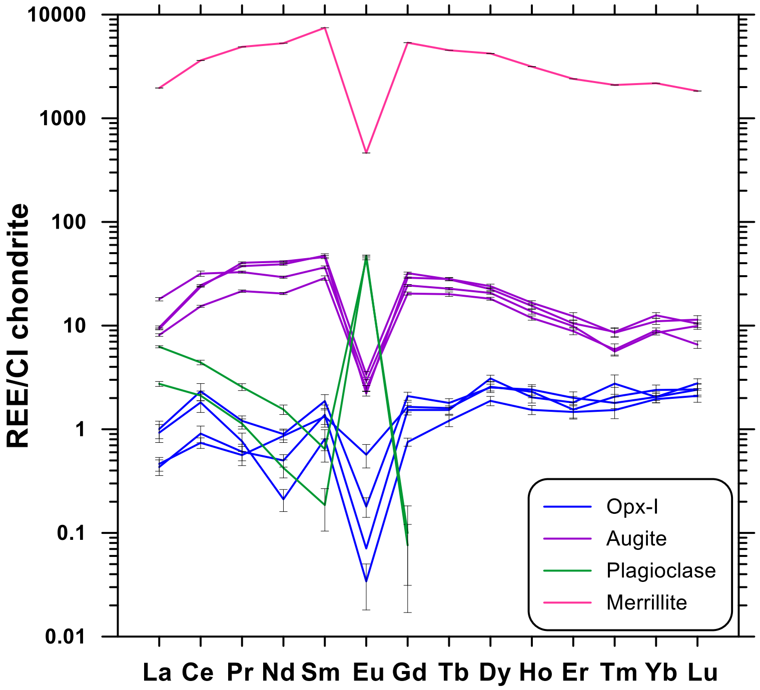

Supplementary Fig. 4 | Chondrite-normalized rare earth element patterns in orthopyroxene (Opx-I), augite, plagioclase, and merrillite from NWA 8321. Reference CI chondrite data is from Supplementary Ref. 2. The error bars represent 1 standard deviation (SD).

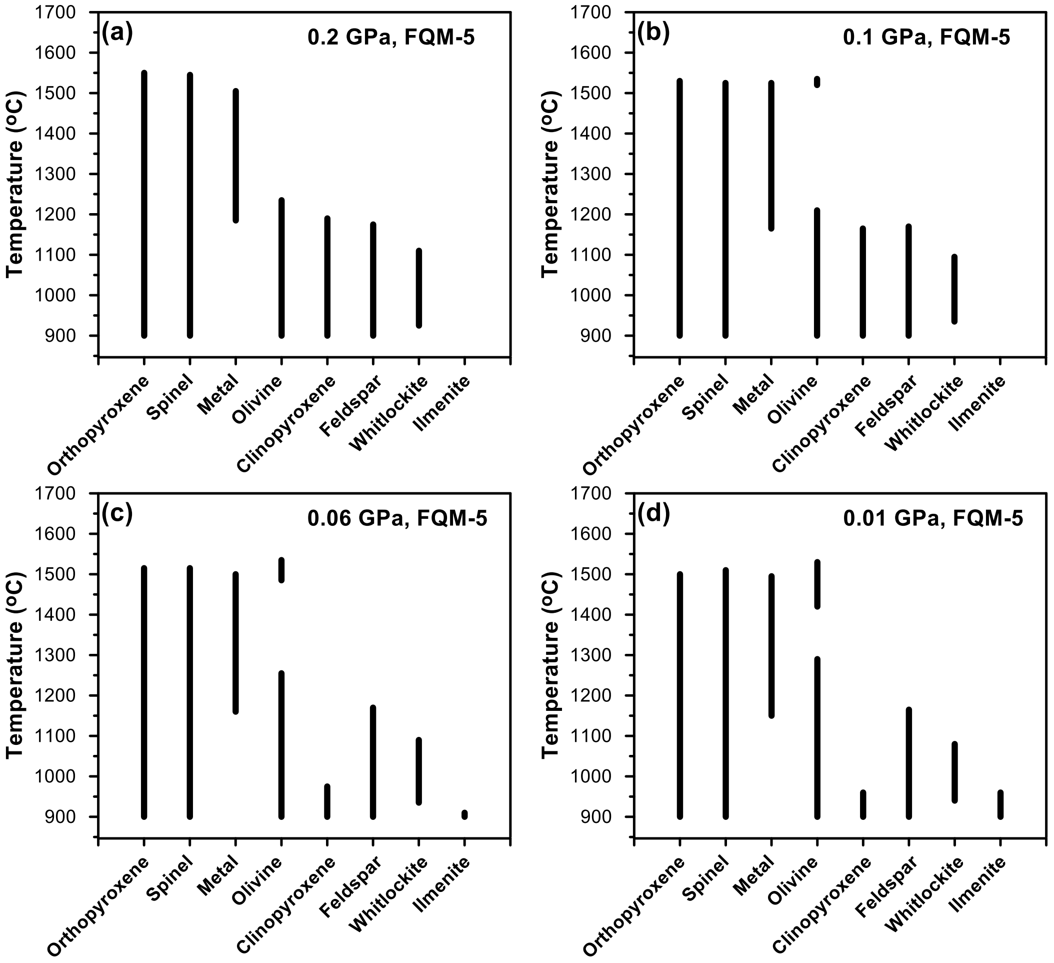

Supplementary Fig. 5 | Representative results of Rhyolite-MELTS calculations at various pressures (0.2 GPa, 0.1 GPa, 0.06 GPa, and 0.01 GPa) and an oxygen fugacity of FMQ-5.

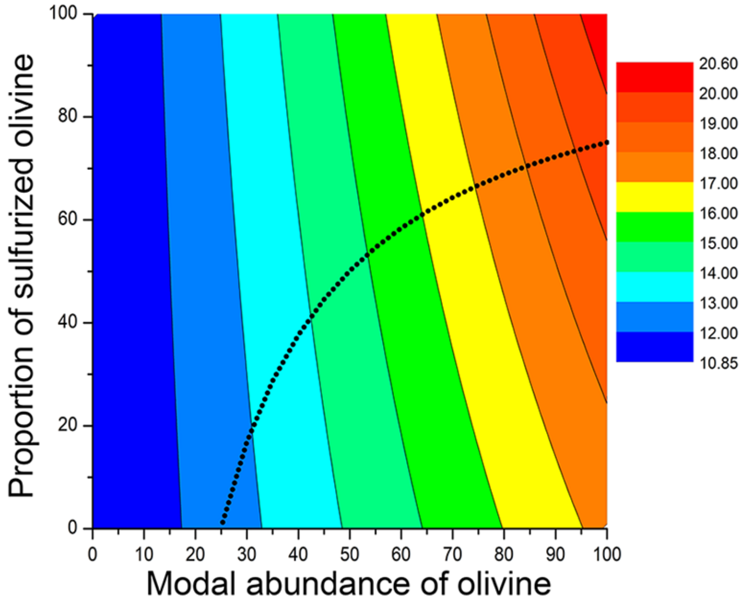

Supplementary Fig. 6 | Distribution of bulk Fe concentrations (wt%) with varying modal abundances of olivine and proportions of sulfurized olivine. The calculation assumes that the olivine-rich lithology (dunite and harzburgite) are composed of orthopyroxene and olivine. The compositions of olivine and Opx-I are selected as their initial compositions. Different colors denote various bulk Fe concentrations. The dotted line denotes the critical proportion of sulfurized olivine to keep the modal abundance of olivine below 25%, which is the minimum abundance for olivine to be detected by the Dawn VIS spectrometer. Above this dotted line, the modal abundance of olivine would be below 25%.

Supplementary Table 1 | Microprobe compositions (wt%) of pyroxene in NWA 8321.

SiO_2	TiO_2	Al_2O_3	Cr_2O_3	MgO	FeO	MnO	CaO	Na_2O	Total	Mg#	Fe/Mn	Wo	En	Fs
Opx-I														
55.6	0.11	2.82	0.95	27.1	12.0	0.47	1.53	0.39	101.0	0.80	24.9	3.1	77.8	19.1
54.6	0.18	0.38	1.58	28.1	12.3	0.45	2.59	bd	100.2	0.80	27.3	5.0	76.4	18.6
54.3	0.14	1.00	0.41	27.7	14.1	0.54	1.62	bd	99.81	0.78	25.9	3.1	75.6	21.3
54.3	0.14	1.13	0.46	27.3	14.3	0.51	1.61	bd	99.75	0.78	27.4	3.2	75.1	21.8
54.5	0.13	1.00	0.45	27.3	14.0	0.55	1.50	bd	99.43	0.78	25.1	3.0	75.5	21.5
55.4	0.14	1.00	0.43	26.5	13.8	0.52	2.66	0.03	100.5	0.78	26.1	5.3	73.4	21.3
55.1	0.14	0.99	0.44	26.6	14.3	0.54	1.64	bd	99.75	0.77	26.2	3.3	74.4	22.3
55.1	0.16	0.95	0.40	27.5	14.2	0.52	1.45	bd	100.3	0.78	26.8	2.9	75.5	21.6
54.2	0.18	1.11	0.35	27.6	14.1	0.52	1.50	0.02	99.58	0.78	26.9	2.9	75.6	21.5
54.6	0.14	0.99	0.32	27.8	13.2	0.51	1.62	0.02	99.2	0.79	25.4	3.2	76.6	20.2
54.4	0.16	0.96	0.29	27.9	13.8	0.52	1.33	0.02	99.38	0.78	26.0	2.6	76.5	20.9
54.1	0.13	0.84	0.32	27.9	14.3	0.54	1.30	0.02	99.45	0.78	26.2	2.5	75.9	21.6
Opx-II														
54.6	0.06	0.51	0.16	29.2	14.0	0.49	0.48	bd	99.50	0.79	28.1	0.9	78.3	20.8
55.4	0.06	0.33	0.08	29.0	14.0	0.53	0.50	bd	99.90	0.79	25.8	1.0	78.1	20.9
55.1	0.04	0.36	0.11	28.9	13.9	0.52	0.46	bd	99.39	0.79	26.6	0.9	78.2	21.0
54.8	0.08	0.38	0.13	28.9	14.0	0.48	0.47	bd	99.24	0.79	28.7	0.9	78.1	21.0
55.2	0.02	0.15	0.02	29.3	14.1	0.50	0.37	bd	99.66	0.79	27.6	0.7	78.4	20.9
54.6	0.08	0.35	0.09	29.2	14.1	0.50	0.46	bd	99.38	0.79	27.8	0.9	78.2	20.9
55.0	0.07	0.34	0.09	29.1	14.1	0.48	0.53	bd	99.71	0.79	28.8	1.0	78.0	21.0
55.2	0.11	0.43	0.15	29.0	13.9	0.49	0.48	0.03	99.79	0.79	28.1	0.9	78.2	20.8
55.0	0.06	0.28	0.06	29.2	14.1	0.52	0.46	bd	99.68	0.79	26.9	0.9	78.1	21.0
54.9	0.05	0.47	0.14	28.9	13.8	0.53	0.49	bd	99.28	0.79	25.7	1.0	78.3	20.8
55.6	0.05	0.44	0.09	29.3	13.9	0.50	0.47	0.07	100.4	0.79	27.4	0.9	78.4	20.7
55.6	0.06	0.35	0.12	29.0	13.8	0.54	0.44	0.03	99.94	0.79	25.0	0.9	78.4	20.7
55.5	0.07	0.42	0.18	28.5	13.6	0.48	0.50	0.02	99.27	0.79	28.1	1.0	78.2	20.8
54.7	0.08	0.53	0.77	28.5	13.8	0.49	0.51	bd	99.38	0.79	27.9	1.0	78.0	21.0
55.8	0.09	0.63	0.17	28.3	14.0	0.49	0.59	0.03	100.1	0.78	28.2	1.1	77.5	21.3
56.1	0.06	0.21	0.08	28.8	14.0	0.53	0.45	bd	100.2	0.79	25.8	0.9	78.1	21.0
55.0	0.13	0.55	1.28	28.6	13.6	0.48	0.36	bd	100.0	0.79	28.3	0.7	78.5	20.8
55.0	0.13	1.57	1.40	28.2	13.2	0.46	0.60	0.04	100.6	0.79	28.1	1.2	78.4	20.4
54.7	0.09	0.33	0.14	29.0	14.1	0.49	0.50	0.03	99.38	0.79	28.5	1.0	78.0	21.1
55.5	0.05	0.36	0.08	29.5	14.3	0.51	0.45	0.03	100.8	0.79	27.8	0.8	78.1	21.0
55.0	0.07	0.47	0.10	29.1	14.0	0.48	0.51	0.02	99.75	0.79	29.1	1.0	78.1	20.9
54.9	0.07	0.37	0.06	28.7	14.2	0.49	0.54	0.03	99.36	0.78	28.6	1.0	77.6	21.4
Augite														
54.1	0.15	0.72	0.37	16.6	4.88	0.28	22.3	0.11	99.51	0.86	17.1	45.1	47.2	7.7

53.5	0.17	0.74	0.62	16.9	5.03	0.24	22.4	0.12	99.72	0.86	20.5	44.7	47.4	7.8
53.7	0.17	0.66	0.28	16.3	4.17	0.20	23.6	0.06	99.14	0.88	21.0	47.6	45.9	6.5
53.7	0.18	0.79	0.56	16.0	5.76	0.30	22.0	0.11	99.4	0.83	19.1	45.0	45.9	9.1
53.9	0.22	1.34	0.63	16.8	5.69	0.28	21.0	0.16	100.0	0.84	20.2	42.9	48.1	9.0
53.5	0.20	1.36	0.64	16.7	5.49	0.30	21.3	0.16	99.65	0.85	17.9	43.5	47.8	8.7
54.0	0.22	1.27	0.53	16.7	5.00	0.29	21.9	0.14	100.1	0.86	17.2	44.5	47.6	7.9
52.9	0.24	1.06	0.47	16.8	4.78	0.27	23.5	0.10	100.1	0.86	17.6	46.3	46.4	7.3
53.2	0.16	0.51	0.28	17.0	4.55	0.23	23.7	0.08	99.71	0.87	20.0	46.5	46.6	7.0
53.3	0.15	0.98	0.92	17.0	4.60	0.24	22.8	0.11	100.1	0.87	19.0	45.5	47.4	7.1
52.6	0.17	0.83	0.46	16.6	5.11	0.29	22.6	0.11	98.77	0.85	17.4	45.4	46.7	8.0
52.4	0.22	1.15	0.72	16.4	4.44	0.26	23.5	0.10	99.19	0.87	16.6	47.2	45.9	6.9
53.2	0.16	0.90	0.82	17.0	4.74	0.24	22.8	0.11	99.97	0.87	19.4	45.4	47.3	7.3
53.0	0.19	1.00	0.41	16.8	4.55	0.24	23.0	0.13	99.32	0.87	18.8	46.0	46.9	7.1
53.6	0.12	0.40	0.13	16.8	4.50	0.19	24.2	0.09	100.0	0.87	24.0	47.3	45.9	6.8

Mg#=Mg/(Mg+Fe) in mole. Bd: below detection limit. Wo=100*Ca/(Mg+Fe+Ca) in mole; En=100*Mg/(Mg+Fe+Ca) in mole; Fs=100*Fe/(Mg+Fe+Ca) in mole.

Supplementary Table 2 | Microprobe compositions (wt%) of plagioclase in NWA 8321

P_2O_5	SiO_2	TiO_2	Al_2O_3	MgO	FeO	CaO	Na_2O	K_2O	Total	An
0.02	44.4	bd	36.3	0.02	0.22	18.2	1.16	0.02	100.3	89.6
0.03	44.3	bd	36.9	0.02	0.27	18.6	0.93	0.02	101.1	91.6
0.05	45.3	0.03	36.3	0.02	0.25	18.2	1.24	0.03	101.4	88.9
0.05	45.1	0.03	36.2	bd	0.27	18.1	1.16	bd	100.9	89.6
0.02	45.3	0.03	35.5	0.02	0.07	17.8	1.39	0.03	100.2	87.5
0.02	45.3	bd	35.9	bd	0.08	17.9	1.29	0.04	100.5	88.3

Bd: below detection limit. An=100*Ca/(Ca+Na+K) in mole.

Supplementary Table 3 | Microprobe compositions (wt%) of olivine in NWA 8321

SiO_2	Cr_2O_3	MgO	FeO	MnO	Total	Mg#	Fe/Mn
37.6	bd	39.4	22.3	0.44	99.74	0.76	50.3
37.6	bd	39.9	21.6	0.44	99.54	0.77	48.7
37.8	0.02	39.3	22.0	0.47	99.59	0.76	46.0
38.3	bd	40.3	21.7	0.41	100.7	0.77	51.8
38.4	0.05	39.9	21.8	0.45	100.6	0.77	48.4
38.7	0.03	40.1	21.5	0.44	100.8	0.77	47.7
38.6	bd	39.4	22.0	0.46	100.5	0.76	47.4
38.3	bd	39.4	22.4	0.44	100.5	0.76	50.5
38.4	0.15	39.4	21.9	0.44	100.3	0.76	49.7
38.5	0.12	39.7	21.9	0.48	100.7	0.77	44.7
38.2	0.04	40.0	21.1	0.44	99.78	0.77	47.0
38.2	0.05	39.3	21.4	0.4	99.35	0.77	52.6
38.0	bd	39.6	21.8	0.41	99.81	0.77	52.4
37.8	0.02	39.6	22.0	0.47	99.89	0.76	46.1

Mg#=Mg/(Mg+Fe) in mole. Bd: below detection limit. Fo=100*Mg/(Mg+Fe) in mole.

Supplementary Table 4 | Microprobe compositions (wt%) of chromite in NWA 8321

TiO_2	Al_2O_3	Cr_2O_3	MgO	FeO	MnO	Total	Mg#	Chr	Spl	Usp
Coarse-grained chromite										
1.06	16.4	49.2	4.65	28.6	0.67	100.6	0.23	65.0	32.3	2.7
1.00	16.8	49.3	4.53	28.6	0.64	100.9	0.22	64.7	32.8	2.5
0.97	17.5	47.6	4.81	28.0	0.61	99.49	0.24	63.0	34.6	2.4
1.06	17.1	48.3	4.67	28.2	0.62	99.95	0.23	63.7	33.6	2.6
Fine-grained chromite										
0.63	13.5	51.9	3.39	29.5	0.72	99.64	0.17	70.9	27.5	1.6
0.61	12.8	52.5	3.13	29.1	0.68	98.82	0.16	72.1	26.3	1.6
0.57	12.7	52.8	2.77	29.5	0.66	99.00	0.14	72.6	26.0	1.5
0.99	17.1	49.2	4.17	28.5	0.61	100.6	0.21	64.3	33.3	2.5
0.75	17.2	49.4	4.41	28.3	0.55	100.6	0.22	64.7	33.5	1.9
1.00	16.0	50.5	4.65	28.0	0.63	100.8	0.23	66.3	31.2	2.5
0.76	15.9	51.1	4.14	28.4	0.61	100.9	0.21	67.0	31.1	1.9

Mg#=Mg/(Mg+Fe) in mole. Chr=100*Cr/(Cr+2*Ti+Al) in mole; Spl=100*Al/(Cr+2*Ti+Al) in mole; Usp=100*2*Ti/(Cr+2*Ti+Al) in mole.

Supplementary Table 5 | Microprobe compositions (wt%) of merrillite in NWA 8321

P_2O_5	SiO_2	Cr_2O_3	MgO	FeO	MnO	CaO	Na_2O	K_2O	Total
46.5	0.06	0.25	3.46	0.82	0.03	46.1	2.11	0.07	99.40
46.2	bd	0.03	3.48	0.97	bd	45.6	2.36	0.08	98.72
45.5	0.08	bd	3.62	0.84	bd	48.0	2.26	bd	100.3
45.0	0.65	0.32	3.72	0.69	0.04	47.8	2.10	bd	100.3

Bd: below detection limit.

Supplementary Table 6 | REE compositions (ppm) of minerals in NWA 8321

	Orthopyroxene (Opx-I)								Augite			
	4OPX	1SD	5OPX	1SD	9OPX	1SD	7OPX	1SD	8AUG	1SD	3AUG	1SD
La	0.10	0.02	0.11	0.02	0.24	0.05	0.22	0.04	2.25	0.08	2.19	0.05
Ce	0.55	0.10	0.45	0.05	1.40	0.26	1.10	0.22	14.79	0.26	14.28	0.16
Pr	0.05	0.01	0.06	0.01	0.11	0.01	0.07	0.01	3.34	0.04	3.60	0.07
Nd	0.23	0.03	0.39	0.05	0.41	0.05	0.10	0.02	17.67	0.39	18.77	0.29
Sm	0.20	0.05	0.23	0.03	0.28	0.04	0.12	0.03	6.98	0.27	6.75	0.24
Eu	0.01	0.01	0.03	0.01	0.01	0.01	0.01	0.01	0.19	0.01	0.12	0.01
Gd	0.30	0.03	0.36	0.03	0.41	0.04	0.15	0.01	6.30	0.15	5.70	0.08
Tb	0.06	0.01	0.06	0.01	0.07	0.01	0.04	0.01	1.01	0.03	1.02	0.04
Dy	0.75	0.05	0.62	0.08	0.61	0.05	0.46	0.05	5.45	0.17	5.82	0.27
Ho	0.11	0.01	0.13	0.02	0.13	0.02	0.09	0.01	0.86	0.05	0.92	0.04
Er	0.29	0.04	0.25	0.04	0.32	0.05	0.23	0.03	1.67	0.13	1.95	0.17
Tm	0.07	0.01	0.05	0.01	0.04	0.01	0.04	0.01	0.21	0.02	0.21	0.02
Yb	0.33	0.04	0.39	0.05	0.33	0.03	0.32	0.03	2.04	0.13	1.79	0.12
Lu	0.06	0.01	0.06	0.01	0.07	0.01	0.05	0.01	0.25	0.02	0.28	0.03

	Augite				Plagioclase				Merrillite	
	4AUG	1SD	5AUG	1SD	8AN	1SD	9AN	1SD	4MRL	1SD
La	4.22	0.16	1.90	0.06	0.64	0.04	1.47	0.03	459	4
Ce	19.13	1.14	9.26	0.21	1.27	0.14	2.66	0.13	2180	16
Pr	2.92	0.07	1.92	0.05	0.10	0.01	0.23	0.02	436	3
Nd	13.25	0.32	9.23	0.22	0.19	0.04	0.70	0.07	2396	23
Sm	5.37	0.17	4.23	0.21	0.03	0.01	0.09	0.02	1097	13
Eu	0.16	0.01	0.14	0.01	2.65	0.03	2.47	0.05	26	1
Gd	4.80	0.11	4.01	0.14	0.01	0.01	0.02	0.02	1054	19
Tb	0.82	0.02	0.73	0.04					164	2
Dy	5.01	0.14	4.40	0.15					1024	13
Ho	0.76	0.04	0.66	0.03					175	3
Er	1.57	0.13	1.40	0.11					381	12
Tm	0.14	0.01	0.14	0.02					51	2
Yb	1.39	0.07	1.46	0.08					354	6
Lu	0.24	0.02	0.16	0.01					45	1

Supplementary Table 7 | Whole-rock compositions (wt%) of major elements in NWA 8321

P_2O_5	SiO_2	TiO_2	Al_2O_3	Fe_2O_3(tot)	Cr_2O_3	MnO	MgO	CaO	Na_2O	K_2O	Total
0.03	51.24	0.14	4.02	15.77	0.97	0.44	23.78	3.10	0.07	0.01	99.56

Supplementary References

1. Li, S., Yin, Q. Z., Bao, H., Sanborn, M. E., Irving, A., Ziegler, K., Agee, C., Marti, K., Miao, B., Li, X., Li, Y. & Wang S. Evidence for a multilayered internal structure of the chondritic acapulcoite-lodranite parent asteroid. *Geochimica et Cosmochimica Acta* **242**, 82–101 (2018).
2. Anders, E. & Grevesse, N. Abundances of the elements-Meteoritics and solar. *Geochimica et Cosmochimica Acta* **53**, 197–214 (1989).
